# RADAR-Base: Open Source Mobile Health Platform for Collecting, Monitoring, and Analyzing Data Using Sensors, Wearables, and Mobile Devices

**DOI:** 10.2196/11734

**Published:** 2019-08-01

**Authors:** Yatharth Ranjan, Zulqarnain Rashid, Callum Stewart, Pauline Conde, Mark Begale, Denny Verbeeck, Sebastian Boettcher, The Hyve, Richard Dobson, Amos Folarin, The RADAR-CNS Consortium

**Affiliations:** 1 The Institute of Psychiatry, Psychology & Neuroscience (IoPPN) Department of Biostatistics & Health Informatics King's College London London United Kingdom; 2 Vibrent Health Fairfax, VA United States; 3 Janssen Pharmaceutica NV, Turnhoutseweg Beerse Belgium; 4 Epilepsy Center, Department of Neurosurgery, University of Hospital Freiburg Freiburg Germany; 5 The Hyve MJ Utrecht Netherlands; 6 Institute of Health Informatics, University College London London United Kingdom; 7 The RADAR-CNS Consortium London United Kingdom

**Keywords:** remote sensing technology, mobile applications, telemedicine, mental health

## Abstract

**Background:**

With a wide range of use cases in both research and clinical domains, collecting continuous mobile health (mHealth) streaming data from multiple sources in a secure, highly scalable, and extensible platform is of high interest to the open source mHealth community. The European Union Innovative Medicines Initiative Remote Assessment of Disease and Relapse-Central Nervous System (RADAR-CNS) program is an exemplary project with the requirements to support the collection of high-resolution data at scale; as such, the Remote Assessment of Disease and Relapse (RADAR)-base platform is designed to meet these needs and additionally facilitate a new generation of mHealth projects in this nascent field.

**Objective:**

Wide-bandwidth networks, smartphone penetrance, and wearable sensors offer new possibilities for collecting near-real-time high-resolution datasets from large numbers of participants. The aim of this study was to build a platform that would cater for large-scale data collection for remote monitoring initiatives. Key criteria are around scalability, extensibility, security, and privacy.

**Methods:**

RADAR-base is developed as a modular application; the backend is built on a backbone of the highly successful Confluent/Apache Kafka framework for streaming data. To facilitate scaling and ease of deployment, we use Docker containers to package the components of the platform. RADAR-base provides 2 main mobile apps for data collection, a Passive App and an Active App. Other third-Party Apps and sensors are easily integrated into the platform. Management user interfaces to support data collection and enrolment are also provided.

**Results:**

General principles of the platform components and design of RADAR-base are presented here, with examples of the types of data currently being collected from devices used in RADAR-CNS projects: Multiple Sclerosis, Epilepsy, and Depression cohorts.

**Conclusions:**

RADAR-base is a fully functional, remote data collection platform built around Confluent/Apache Kafka and provides off-the-shelf components for projects interested in collecting mHealth datasets at scale.

## Introduction

### Background

The opportunity in health care for continuous monitoring of patients has steadily grown in parallel with the widespread availability of smartphones, more capacious mobile networks, and development of new wearable sensors that are able to continuously measure a growing set of physiological and phenomenological parameters. Many of these devices are currently in the lifestyle or fitness domain; however, vendors are increasingly developing these devices for medical-grade applications. If these streams of data can be reliably collected, analyzed, and acted on, it opens up the possibility of better understanding disease etiology, diagnosis, prognosis, and detecting relapse in most disease areas.

Existing mobile health (mHealth) platforms include some form of questionnaires, phone (Android, iOS) sensor data collection, wearables integration, backend infrastructure, and management user interface, but few presently include all or are scalable solutions. Moreover, 2 such examples are the Open source AWARE Framework, an Android platform for mobile phone–based context sensing [1], and the Health Insurance Portability and Accountability Act of 1996-compliant Bridge platform, which supports biomedical studies conducted through smartphones [2].

### Objectives

The Euro 26 million Innovative Medicines Initiative (IMI) Remote Assessment of Disease and Relapse -Central Nervous System (RADAR-CNS) is a major international academic-industry research program aimed at developing novel methods and infrastructure for monitoring major depressive disorder (MDD), epilepsy (Epi), and multiple sclerosis (MS) using wearable devices and smartphone technology [3]. Beyond supporting the initial goals of the 3 disorder areas in RADAR-CNS, the RADAR-base platform aims to provide a highly extensible platform for mHealth applications [4].

To facilitate adoption by the wider mHealth community, the RADAR-base platform was released under an open source Apache 2 license in January 2018. RADAR-base is composed of backend infrastructure and 2 Android mobile apps: a cross-platform Cordova app for active monitoring of participants (active remote monitoring technology, aRMT) through conscious action (eg, questionnaires, audio questions, timed tests) and a native Android app for passive monitoring via phone and wearable sensors (passive remote monitoring technology, pRMT). RADAR-base also includes capabilities for data aggregation, management of studies, and real-time visualizations.

A key differentiator of RADAR-base platform is that it makes use of Confluent technologies (based around Apache Kafka) to provide an end-to-end solution for remote monitoring use cases (eg, participant management and data analysis) that scales horizontally through the use of Kafka and Confluent ecosystem. Other approaches to centralize information flow and decouple systems exist, in particular Messaging Queues and Enterprise Service Bus/Service Oriented Architecture (SOA) type architectures, which have a number of overlapping and differentiating factors compared with Confluent/Kafka, in particular around routing, scaling, performance, and ecosystem [5,6]. Kafka and the Confluent ecosystem have become the de facto industry standard for high-throughput event streaming data applications. More specifically, it is appropriate for building real-time streaming/transforming data pipelines that reliably move data between systems at scale. The RADAR-base platform has a number of requirements: first, data must flow from sensors or data sources into the platform through mobile devices and second, to transform these data (eg, restructured for Cold Storage, Hot Storage, aggregating data based on time windows and monitor the data coming in. This Kafka based pipeline, along with data schematization makes the platform flexible and open to a variety of devices, types of data, velocity, and throughput.

The RADAR-base platform can be deployed both in local settings, such as a hospital, for local data collection or for ambulatory studies through remote deployment for centralized data collection. The RADAR-base backend has been deployed on various platforms (cloud, bare-metal, etc) as a set of microservices using Docker containers. Both scenarios are used in RADAR-CNS.

The RADAR-base platform is a scalable, secure, open source Internet of Things (IoT) platform for real-time remote sensor data collection in the context of mHealth clinical studies.

## Methods

### Components

The RADAR-base platform consists of following major categories of components:

1. Data Collection Ecosystem
2. Data sources
3. Data Processing and Visualization
4. Study management and Security

**Figure 1:**
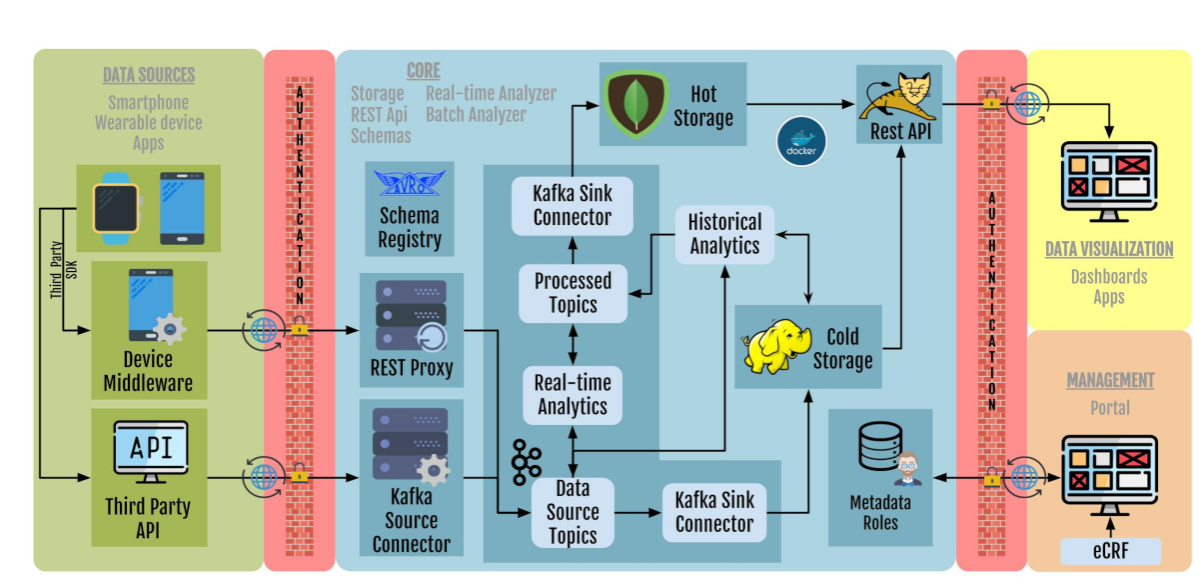
Technical overview of the RADAR-base platform stack.

**Figure 2:**
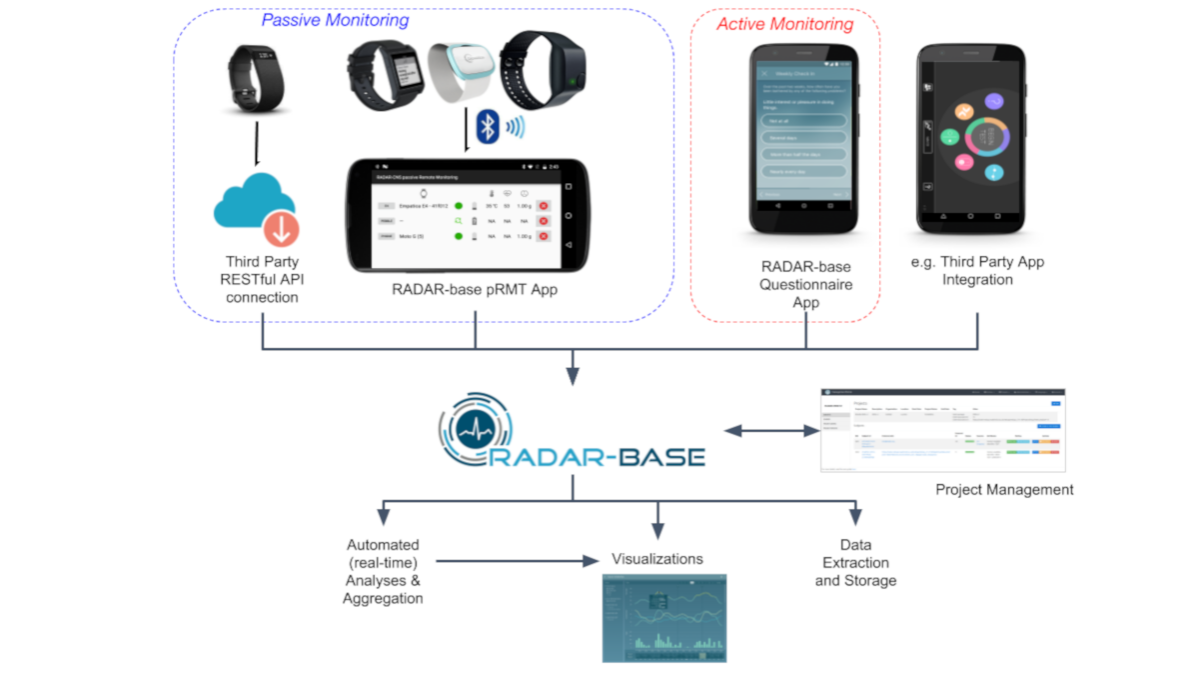
Current data sources: Empatica E4, Pebble 2, Fitbit, Biovotion, Faros, active Remote Monitoring Questionnaire app, and passive Remote Monitoring app.

Figures 1 and 2 show the integration and communication between different components of the RADAR-base platform.

Figure 1 shows the Technical Overview of the RADAR-base Platform Stack Platform Capabilities:

1. High throughput, low latency data collection
2. Scalability
3. Generalized device integration for passive data sources
  1. Abstracted and composable integration mechanism for sensor devices
  2. Third-Party RESTful (Representational State Transfer) data source integration
4. Configurable data sources at runtime
5. Schema evolution
6. Real-time data processing and analytics
7. Hot and cold storage
8. Data access (Representational State Transfer [REST]-API)
9. Modular, extensible dashboards
10. Electronic Case Report Form (eCRF) integration (REDCap)
11. Remote configuration
12. Cohort Management Portal
13. Security

Figure 2 shows an overview of the RADAR-base platform. Current data sources are as follows: Empatica E4, Pebble 2, Fitbit, Biovotion, Faros, Active aRMT Questionnaire app, and Passive pRMT app.

These functionalities are delivered through the following components:

1. Data ingestion: Recognizing and registering data sources (including smartphones and wearable devices), collecting the data via a direct Bluetooth connection or through a third-party application protocol interface (API), and streaming in near-real-time to the server (green box in Figure 1). Using Apache Kafka, the collected data are streamed to dedicated topics in real-time where the data are schematized using Apache AVRO and the Schema Registry. More details about data source mapping and integration can be found in the Data Sources and Study Management Sections below. Detailed further on the platform's documentation wiki [7].
2. Data storage and management: Consists of 2 centralized storage systems behind an authorized security layer. The cold storage, based on Hadoop Distributed File System (HDFS), that is scalable and fault-tolerant, focused on storing large volumes of raw data, and the hot storage, based on MongoDB, for storing aggregated data to provide a near real-time overview of the raw data, principally for the data dashboards.
3. Data sharing: Visualizing aggregated data in a live dashboard and exporting raw data for further analyses in various formats including AVRO, JSON, and CSV.

### Data Collection Ecosystem

The entire RADAR-base backend is deployable as a set of microservices based on Docker containers [8].

#### Representational State Transfer Proxy

As the platform is based on Apache Kafka, we can either send data directly into Kafka via a native Producer or using HTTP and the Confluent REST proxy.

#### Schema Registry

The data sent into the platform from the data source will be converted into AVRO format before going into Kafka topics. To convert our data to AVRO, the REST proxy needs to know the schema (or format) of the data being sent. These schemas are stored in the Schema Registry to reduce the payload size of each request.

#### Backend Streams and Monitors

Event-by-event stream processing is built on top of Kafka Streams. It provides an abstract layer to monitor and analyze streams of data and write aggregated/transformed data into Kafka topics. The data are *produced* (into Processed Topics) and *consumed* (from Data Source Topics) in Apache Avro format using the schema stored inside the Schema Registry. This capability provides real-time analytics and is used to trigger real-time interventions (eg, providing real-time data source statistics and sending notifications on source disconnection).

#### Sink Connectors

The data coming into Kafka is extracted into storage systems such as the HDFS and MongoDB with the help of Kafka Sink Connectors. The HDFS Sink Connector takes raw data coming into the system and deposits it into the HDFS storage in AVRO format (this time with the schema embedded, so the data are self-describing), which can be used for archival storage and historical analysis. The MongoDB sink connector takes aggregated data coming into Kafka from the Streams app’s Processed Topics and deposits it into the MongoDB storage.

#### Data Typing

The AVRO schema is a JSON format specification of the fields and data types, which data values can hold. The schema itself can be embedded in the message or a reference held to the schema stored in a Schema Registry. AVRO is a particularly convenient format for managing schema evolution in RADAR-base, as schema changes can occur frequently and without warning, especially where third-party data sources are concerned.

**Figure 3:**
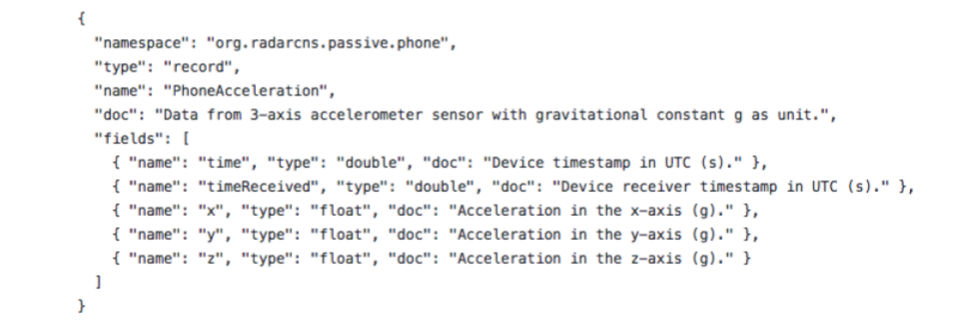
Schema overview for the phone acceleration.

For illustration purposes, the schema for the phone acceleration is shown in Figure 3.

### Data Sources

Data Sources represent a wide variety of systems able to send data into the RADAR-base platform; these include devices containing sensors, mobile phones, questionnaires and digital games/assessments, and Web-APIs data portals.

In RADAR-base, passive data sources are collected via the pRMT app, active data sources via the aRMT app, and third-party data sources via the THINC-it app in RADAR-CNS.

Another type of data source includes middleware connecting a vendor’s Web API to the RADAR-base platform. For example, Fitbit does not provide a mobile Software Development Kit (SDK) to stream data to the pRMT app directly; instead, all the data are uploaded to the vendor data warehouse and provided to developers via a Web API. Getting these data into the RADAR system is achieved by implementing a server-side Kafka Source Connector, which continuously queries data from the vendor’s Web API and dumps it into Kafka inside the RADAR-base platform; this approach can be used to integrate other Web API/OAuth2 data sources [9].

#### Passive Remote Monitoring App: Sensor Data Collection

The native passive Android application (pRMT) has been designed to passively collect data from sensors on the user’s smartphone as well as to integrate wearable devices that offer SDKs. Its enhanced modularity (via pRMT plugins) allows easy integration of new devices/sensors. It currently supports Empatica E4 Wristband, Pebble 2 Smartwatch, and Biovotion VSM devices.

**Figure 4:**
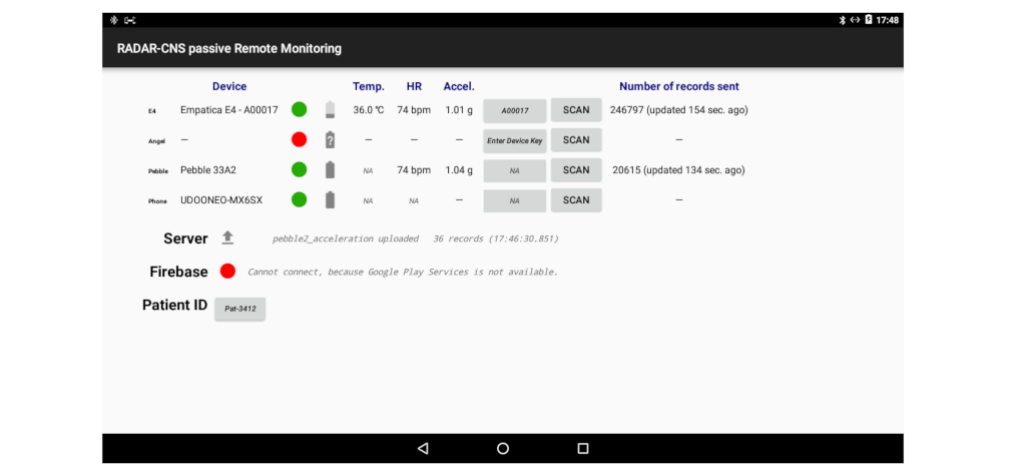
Passive Remote Monitoring app user interface. The Device column lists all the devices that are connected to the app and collect data. Device connection/disconnection is shown by green and red icons, respectively. The 3 columns next to “Device show the different values that are being measured on the connected devices. The last column shows the amount of data (or records) that have been collected.

Figure 4 shows the pRMT app interface after login.

#### Active Remote Monitoring App: Composable Questionnaire Delivery

The primary goal for the aRMT mobile app is to allow users to submit questionnaires through the user’s smartphone at a notified time. The questionnaire definitions and their regimen are defined by simple JSON configuration files, which are Web-served and therefore remotely configurable. New questionnaire configuration files can be easily created either manually or authored as REDCap data dictionaries and parsed via a simple script. The regimen or protocol configuration file defines the sequence of the questionnaires delivered and the local notifications or Firebase Cloud Messaging push notifications used to alert the user.

**Figure 5:**
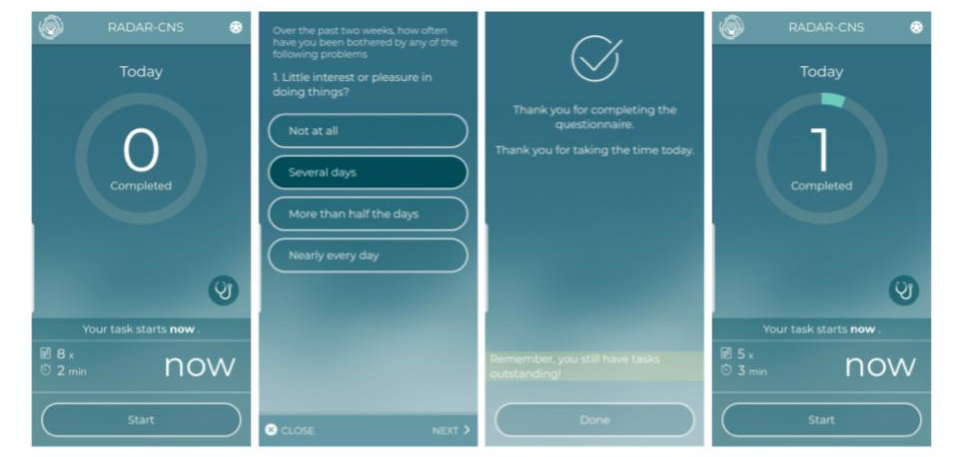
User interface of the active Remote Monitoring app.

The aRMT app was designed as a hybrid Cordova app and usable on both iOS and Android. Furthermore, it includes Cordova plugins to collect active audio responses to questions, allowing active samples of raw speech audio to be collected for analysis. Finally, the aRMT app also serves as a means of providing time markers to data collected in parallel by the pRMT app, such as start and end labels of walking and balance tests used in the MS study in RADAR-CNS. Figure 5 shows a selected interface of the aRMT app.

#### Third-Party App Integration: THINC-it

THINC-it is a third-party mobile app that makes use of 5 quick interactive tests to assess memory, concentration, and attention [10].

The THINC-it app uses the RADAR-base platform backend infrastructure as part of the RADAR-CNS project. It also provides a reasonable paradigm for other third-party app integration into the RADAR-base platform.

### Data Processing and Visualization

#### Historic Raw Data Processing and Visualization

**Figure 6:**
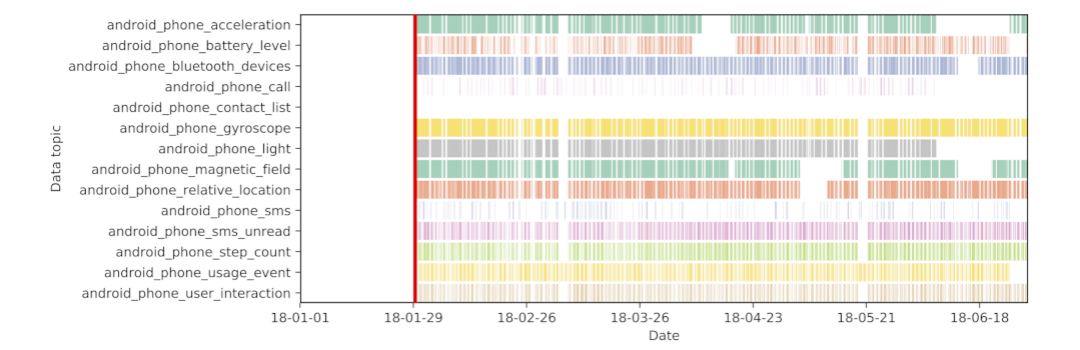
Contiguity of phone sensor data over 6 months collected through RADAR-base for aparticipant in the major depressive disorder study. The red line corresponds to the enrollment date, whereas a coloured segment on each row corresponds to recorded data at an hourly resolution.

A common task is the exploration of collected raw data. In addition to the near-real-time visualization through the dashboard, the RADAR platform includes a python package for the processing and visualization of historic data. The package provides standard tooling for exploratory visualization of RADAR-base data (see Figure 6) and the easy implementation of preprocessing pipelines to take data exported from a RADAR-base project and output the processed data, with any accompanying labels, in a format suitable for use in standard machine learning libraries. Dask, a python library for parallel and larger-than-memory computing [11], is primarily used as the backbone. The potential for large longitudinal studies collecting high-frequency data necessitates the ability to distribute computation or to work on subsets. Moreover, the use of existing libraries allows straightforward integration with the rest of the python data science ecosystem.

The package can make use of the common structure of RADAR-base data through the defined AVRO *schema* and *specification* for each modality. A schema describes data types, allowing automatic parsing of modalities stored without type. Each data source integrated into RADAR also contains a *specification* listing associated sensors with information on each. That information includes field names and units, allowing visualizations to be labeled automatically. As the specifications give the device and type of sensor for the associated data, those data can be mapped to relevant processing pipelines. For example, the Biovotion VSM1 device provides all its data in 1-second batches. These batches are ordered and so can be upsampled. Using the device specification, a function to upsample the data can be mapped to all the relevant data modalities. Alternatively, an analyst may wish to map a function to sensors of the same type across different devices, which is also made simple by using the specification to identify data from sensors of the same type.

#### Real-Time Data Processing and Representational State Transfer (RESTful)Application Protocol Interface

The RADAR platform exposes RESTful Services implemented using Jersey 2 and deployed on Grizzly server. Data collected in the platform are processed in real-time by a Kafka Streams application to provide aggregations (mean, max, etc) at various time resolutions (second, minute, hour, day, and week) and stored in MongoDB, which is served through the REST-API. The REST-API provides various API endpoints, which can be used to request the aggregated data in near-real-time and allows various combinations of queries. All the endpoints are secured; therefore, only a valid user or a client registered with the management portal with appropriate permissions/scopes can access these endpoints. All REST endpoints have been documented following OpenAPI specifications using Swagger: a powerful open source framework offering a large ecosystem of tools that help to design, build, document, and consume RESTful-APIs.

The REST-API also exposes some real-time information about the current status (connected or disconnected) of the sources registered for a particular subject along with when a data source was last detected sending data. All this information is used by the Dashboard to provide a real-time visualization of the current state of the studies/projects to project admins.

#### Real-Time Visualization Via Dashboards

**Figure 7:**
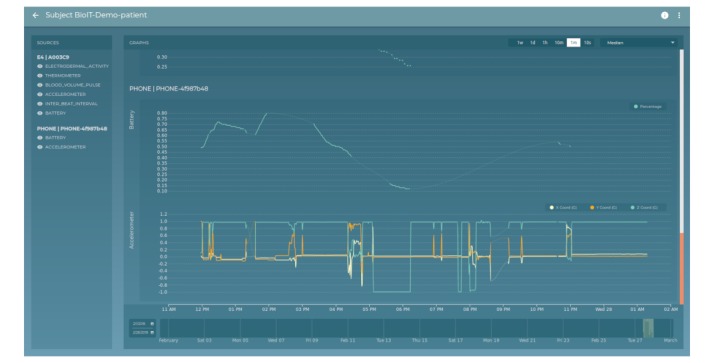
Participant data view (battery and accelerometer streams).

Overview and visualization are provided by a clear, customizable user interface with an emphasis on exploring different aggregation and zoom levels in the data. The RADAR-base dashboards use Angular, RxJS, and D3 to construct views on data from the REST-API. These presently provide management project/study lists, compliance views, and participant-level visualization of longitudinal data. Figure 7 shows an example data view. More detailed figures are given in the Multimedia Appendix 1.

### Study Management and Security

**Figure 8:**
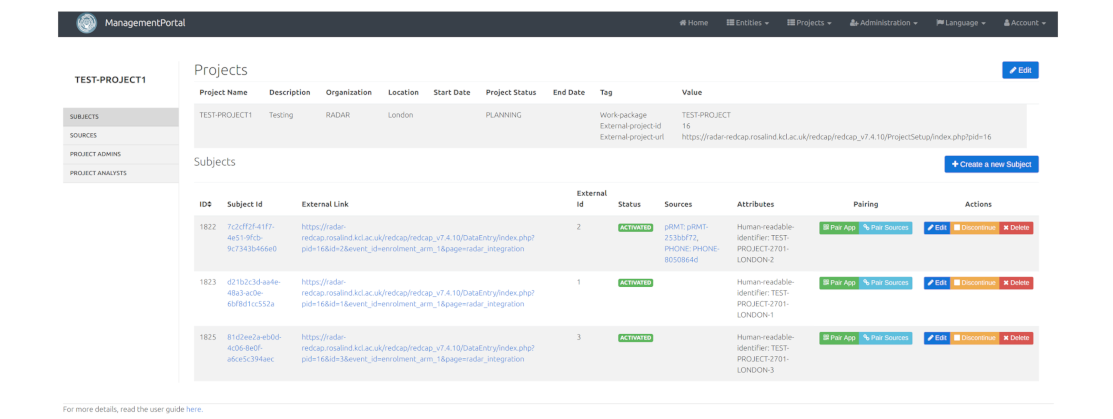
RADAR-base Management Portal.

The management portal Web application is the main user interface for creating and organizing RADAR projects, enrolling participants, and managing the association of participants with corresponding data sources. Figure 8 shows the management portal interface.

Authentication, authorization, deidentification, and encryption are compulsory due to the sensitive information collected by the platform and to manage additional unknown risks associated with IoT using large numbers of network edge devices and endpoints. These are implemented for these following elements:

#### Quick Response Code Authentication

The management portal is used to issue a Quick Response (QR) code or Token for data-source to participant registration. This QR code can be scanned with the embedded QR code scanner in the integrated apps or alternatively a token can be entered directly as text. The decoded QR code provides some valuable information required by clients including sources, user ID, roles, scopes.

#### Open Authentication 2

To provide authorization and authentication, we utilize the OAuth2 workflow, an industry standard protocol for authorization. In the mobile apps, we use the Refresh Token grant type [12], and for other internal clients, we use the client credentials grant type. For instance, the gateway and the REST-API use the latter, whereas the mobile apps use the former.

For the mobile apps, an access token from the management portal is required for authorization across the platform. Without this token, data sources can neither register nor send data into the platform. The management portal provides a Refresh Token in the form of a QR code associated with a subject. This QR code can be scanned by the mobile apps to obtain a URL to a JSON Web Token, which embeds a Refresh Token and the authorization endpoint as a Bearer Token in an HTTP(S) request to obtain a new Refresh-token and Access token pair. This access token can then be used to access and post data according to the resources, roles, and scopes specified. Once the Access token expires, the most recently obtained Refresh Token is used to obtain a new (Refresh-token+Access token) pair.

The RADAR-base platform provides utilities for clients to easily manage the OAuth2 authorization flow [13]. An online manual is available, which specifies a step-by-step process of integrating new apps along with a list of utilities and libraries for QR code and OAuth [14]. The pRMT Android app uses OpenID Connect for authorization and authentication [15].

#### Deidentification

As discussed in eCRF Integration and REDCap Integration WebApp Sections, the strongly identifiable information is saved in a separate eCRF system, which is isolated from the RADAR-base platform [16]. Only the nonidentifiable data are saved in the platform. The 2 separated datasets are linked to each other as pseudonymized data only via RADAR-base Universally Unique ID.

#### Reverse Proxy

An nginx web server is used to proxy traffic into the platform and provide Cross-Origin Resource Sharing. Furthermore, the reverse proxy (nginx web server) can also be configured to act as a mitigation against Distributed Denial‑of‑Service attacks or using it as an HTTP load balancer.

#### Gateway

This component controls access to the Confluent Kafka REST Proxy for posting data from clients to Kafka through HTTP POST requests. It performs authentication and authorization, content validation, and decompression if needed. It also verifies if the access token sent in the HTTP POST request is valid and has the required privileges to perform the POST request for the specified resource, role, and scope.

#### Audit Log

The various components of RADAR-base keep activity logs at levels appropriate for that component. As the management portal keeps track of all study-related information, device assignments, and participant enrollment information, it keeps the most detailed audit logs. Any modification to the management portal database is stored in an audit record. These audit records store the user who made the modification, the time at which the modification was made, as well as the old and new state of the modified entity, allowing the complete history of all study metadata to be tracked or roll back modifications when necessary. Finally, the management portal also logs when, to what application, and for which user access tokens are being granted. It is important to note that this log is only there for the purposes of auditing. Validation of the tokens is not handled by the management portal. Instead, clients can use the management portal’s public key to validate the digital signature embedded in the access token. This way, components in RADAR-base can horizontally scale up, without the need for the management portal to scale up with them just to be able to keep up with validation requests. See Multimedia Appendix 1 for audit log figure.

### REDCap eCRF Integration

Optional integration of one or more REDCap eCRFs servers is provided with RADAR-base. REDCap is a secure 21 CFR Part 11, FISMA, and HIPAA-compliant Web application for building and managing online surveys and databases [17]. When used in this mode, subject creation is linked automatically between the REDCap and RADAR-base.

### User Registration Workflow

**Figure 9:**
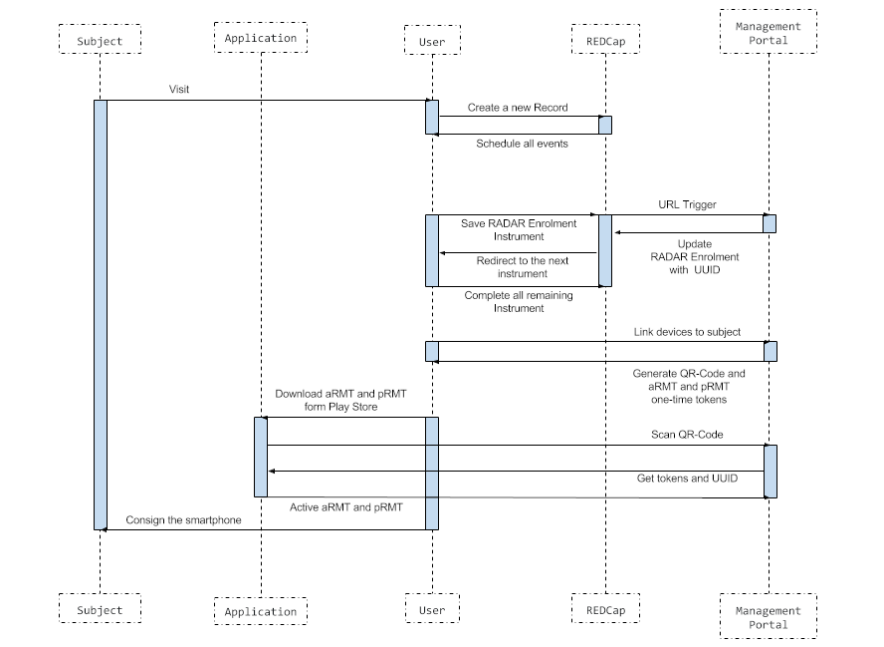
User registration workflow.

A brief workflow of the registration is shown in Figure 9. A subject or recruiter will register a new record in REDcap (optional). Creating this record will trigger the creation of a corresponding subject in the management portal via RESTful-API calls. From the management portal subject, the project admin (recruiter) will be able to register apps via a QR code (aRMT and pRMT). Apps can be downloaded from the playstore, and registration QR codes can be obtained and scanned from the management portal for the particular subject. Once the apps get registered, the relevant subject will start streaming wearables and phone data.

### Software Availability

The entire RADAR-base platform is freely available at github repository as open source software [18]. The details of the platform can be read at the official RADAR-base website [16]. A detailed quickstart, deployment details, and developer documentation are made available at Confluence Wiki [19]. The Docker images for all the components are available at Docker Hub [8].

## Results

Multiple instances of RADAR-base are deployed and in use for real-world studies of Epilepsy, MS, and MDD under the umbrella of RADAR-CNS [20,21].

The catalog of devices currently integrated into the pRMT app include onboard Android smartphone sensors, Empatica E4, Pebble 2 smartwatch, BiovotionEverion, Faros 180, and Fitbit; a list is maintained here [22]. Pluggable capability is provided to integrate new wearable devices offering a native SDK (eg, Empatica E4) or through third-party vendors’ REST-API (eg, Fitbit). The aRMT app provides highly extensible aRMT functionality to the platform, rendering questionnaires from a JSON configuration file, for example, questionnaires using RADAR-CNS include RSES, PHQ8, and ESM. Figure 6 shows the contiguity of data collected from an MDD study participant’s phone over a 6-month period. There are daily patterns visible even at this resolution; topics that rely on user interaction are typically not collected during the night, creating a striped pattern in the figure.

## Discussion

### Remote Assessment of Disease and Relapse-Base Current Deployments

The current deployments of RADAR-base for the different disorders are explained below.

#### Major Depression Disorder

The RADAR-base platform has been deployed centrally to collect active (questionnaires) and passively generated (wearable Fitbit and smartphone sensor) data remotely for participants recruited to 3 sites of the MDD study. The sites include King’s College Hospital, London; Centro de Investigacion Biomedica en Red, Barcelona; and VU University Medical Center, the Netherlands. The objective being to collect regular self-reported symptoms and metrics such as sleep and ambulatory behavior. High-resolution data are being collected over a period of up to 2 years for each participant. More details about the MDD studies and preliminary data analysis are provided in our study [21].

#### Epilepsy

The Radar-base platform has been successfully tested and deployed in the Clinical Neurophysiology Department, King’s College London, and the Epilepsy Center, Medical Center University of Freiburg, in their respective video electroencephalograms monitoring units, and it is currently in active use in London and Freiburg with enrolled participants.

Latest participants have the facility to wear 3 devices (Faros, Biovotion, and E4) concurrently. We have explained the detailed deployment of the platform for Epilepsy studies and initial collected data in our study [20].

#### Multiple Sclerosis

MS studies using RADAR-base are underway at different partner sites across Europe. Participant recruitment has been started and data are being streamed to central deployment. An important focus here is to collect data from the Faros 180 devices used for several mobility and balance tests in addition to similar ambulatory behavior collected in the depression study.

These 3 studies expose the versatility of the RADAR-base platform and generate data with very different complexity, volume, velocity, and durations.

### Technical Challenges

Several technical challenges were addressed, including:

1. High throughput, volume, and velocity of the data.
2. Processing data in real time.
3. Optimizing phone resources to handle data collection and streaming (particularly high-resolution sensors).
4. Privacy concerns particularly around Global Positioning System data used to track location or audio exposing identifiable information. For this, the RADAR-base platform calculates and sends the relative location from a reference point. Similarly, background audio sampling is one-way-mapped to a vector representation of features using an OpenSmile plugin [23]; in this way, raw background audio is not exposed while retaining useful information content for analytics purposes.
5. Identifiable information is kept separate from sensor data to make it pseudonymized.
6. Security (authorization and authentication) is also a major concern for sensitive data collected from participants. Access to data is provided via a secure data transfer protocol.
7. With the huge amount of raw data the platform is built to collect, it is essential to have efficient compression. All the data collected and stored are compressed and encrypted.
8. Maintaining performance and behavior of the data source pRMT and aRMT apps in an ever-changing Android landscape of OS versions, handsets vendors, and form factors.

### Comparison With Prior Work

A summary of other platforms comparing salient features with the RADAR-base platform are provided here. The recently developed mental health Nonintrusive Individual Monitoring Architecture platform, a prototype implementation used alongside an investigation of the key features required of a mHealth data collection platform; these include integrating data sources, a focus on privacy, and flexible user permissions [24]. Intel's Context Sensing SDK is a library for Android and Windows with specific context states; it, however, only provides front-end components [25]. The EmotionSense app is developed by the University of Cambridge to sense emotions with implications for psychological therapy and improving well-being; however, it is only focused on depression [26]. Medopad provides solutions for different health care issues with symptom tracking; this is a commercial solution and mainly focuses on phone sensors and active monitoring methods [27]. PHIT allows users to build health apps based on existing infrastructure [28]. ResearchKit, an open source framework for building apps specifically for iOS, makes it easier to enroll participants and conduct studies. However, new wearable device integration requires strong programming skills and it does not include a data management solution [29].

ResearchStack is an SDK and UX framework for building research study apps on Android, with a similar application domain as ResearchKit [30]. Both ResearchKit and ResearchStack provide software libraries, frameworks, and development tools that require extensive programming skills to create apps. A framework to create observational medical studies for mobile devices without extensive programming skills was presented [31]. Further comparison of currently available sensing platforms/apps is provided in Multimedia Appendix 2.

A key differentiator in the RADAR-base platform is the use of the Confluent platform technologies [32] (based around Apache Kafka) as the underlying infrastructure to provide a highly scalable end-to-end solution for event-driven messaging, which is able to satisfy a wide variety of use cases, for example, high throughput, low latency messaging, real-time data processing, and fault tolerance/robustness. The platform can be deployed as microservices with Docker containers and with minimal effort extended to integrate new sensors and data sources.

### Future Works and Conclusions

RADAR-base aims to stimulate the field of mHealth by providing an off-the-shelf platform for general remote data collection at scale. The project has long-term goals to improve participant care with use cases including predicting and pre-empting relapses and improving outcome measures in trials through the use of remote assessment technologies in a wide variety of disorder areas. Beyond RADAR-CNS, RADAR-base is being deployed across a number of other large EU IMI2–funded programs including RADAR-Alzheimer’s Disease and is presently deployed for BigData@Heart for remote monitoring in an atrial fibrillation treatment trial (the UK National Institute for Health Research—NIHR—funded RATE-AF NCT02391337).

## Appendix

Multimedia Appendix 1 Better resolution figures.

Multimedia Appendix 2 Available sensing platform.

## Abbreviations

API: application protocol interface
aRMT: active remote monitoring technology
CNS: Central Nervous System
HDFS: Hadoop Distributed File System
IMI: Innovative Medicines Initiative
MDD: major depressive disorder
mHealth: mobile health
MS: multiple sclerosis
NIHR: National Institute for Health Research
pRMT: passive remote monitoring technology
RADAR: Remote Assessment of Disease and Relapse
REST: Representational State Transfer
SDK: Software Development Kit
QR: quick response
