# Supplementary material for: RADAR-Base: Open Source Mobile Health Platform for Collecting, Monitoring, and Analyzing Data Using Sensors, Wearables, and Mobile Devices
Source: JMIR Mhealth Uhealth. 2019 Aug 1;7(8):e11734. doi: 10.2196/11734 (PMC6694732; doi:10.2196/11734)
Supplement: Multimedia Appendix 1 [file mhealth_v7i8e11734_app1.pdf]

Supplementary Material

RADAR-base: An Open Source mHealth Platform for Collecting, Monitoring and Analyzing data Using Sensors, Wearables, and Mobile Devices

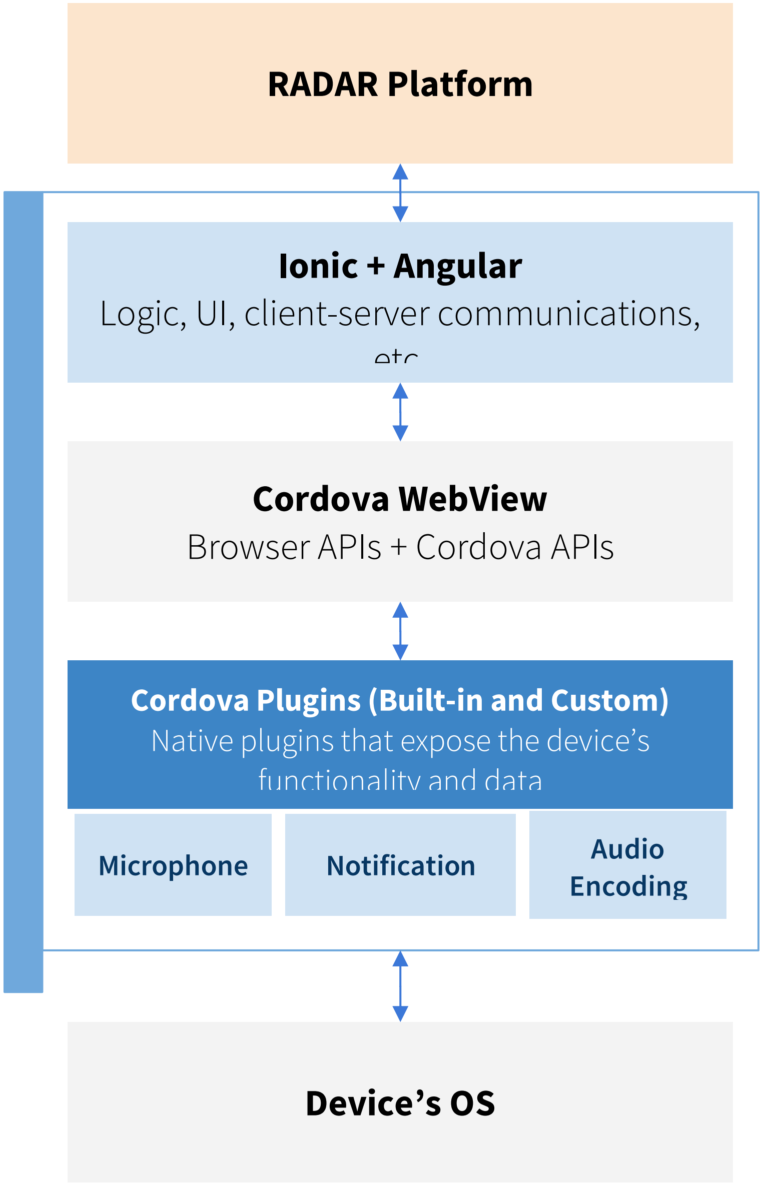

Supplementary Figure 1: Architecture of the aRMT app.

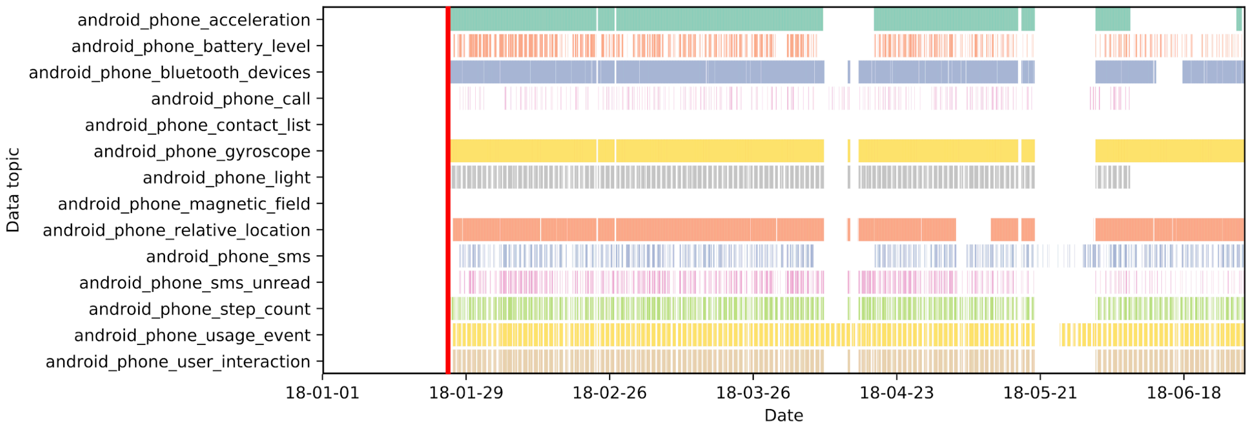

Supplementary Figure 2. Completeness of phone sensor data over 6 months collected through RADAR-base for two participants in the major depressive disorder study. The red line corresponds to the enrollment date, while a coloured segment on each row corresponds to recorded data at an hourly resolution.

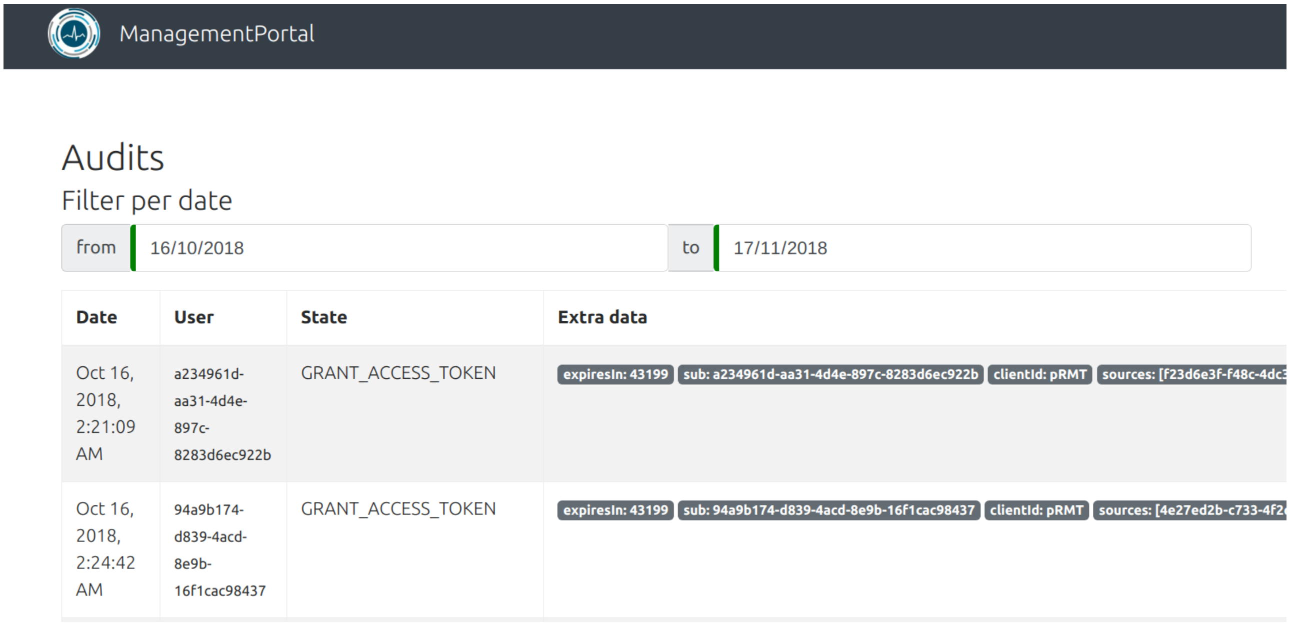

Supplementary Figure 3: Audit logs.

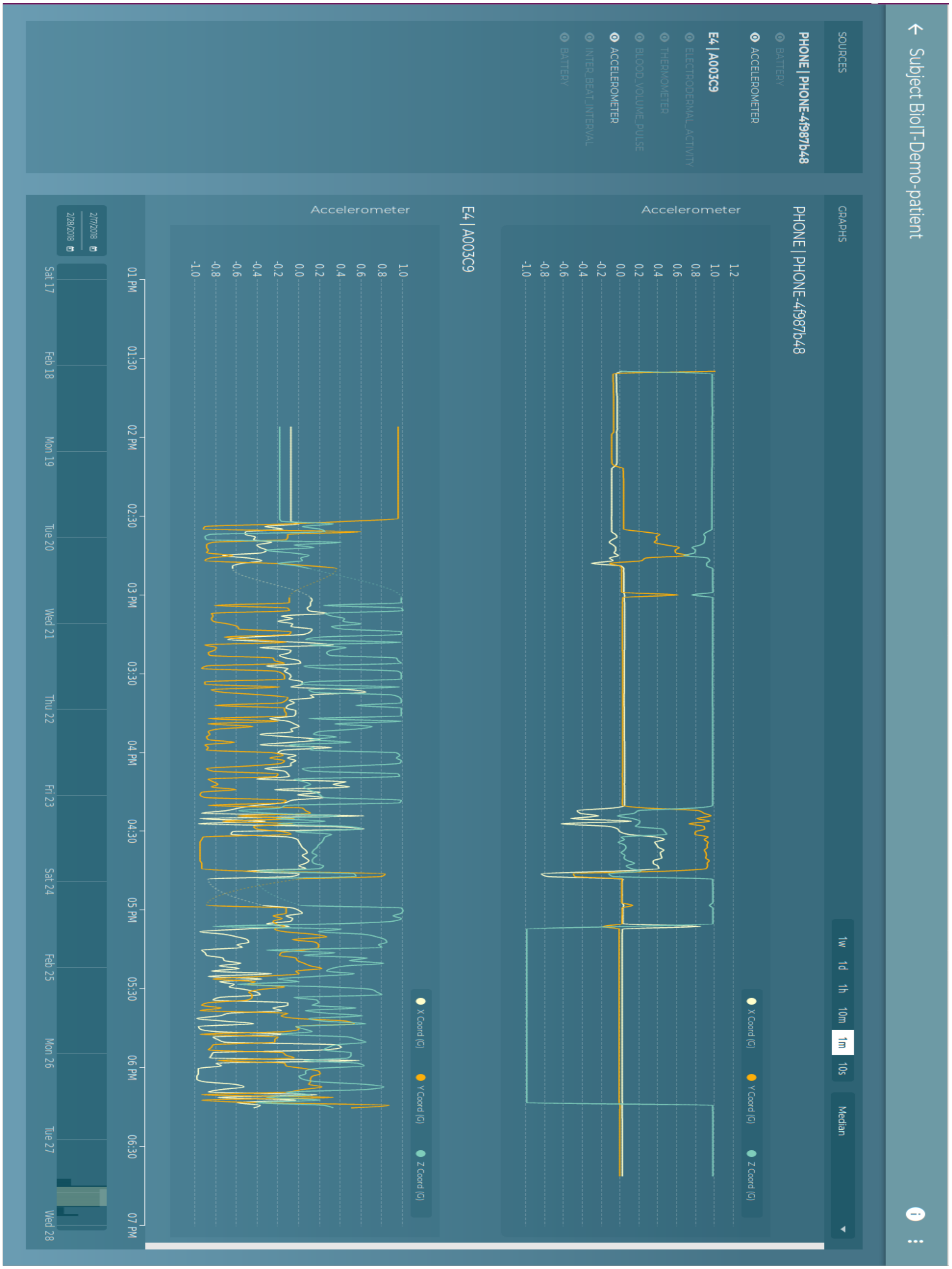

Supplementary Figure 4: Dashboard: Participant’s data view.

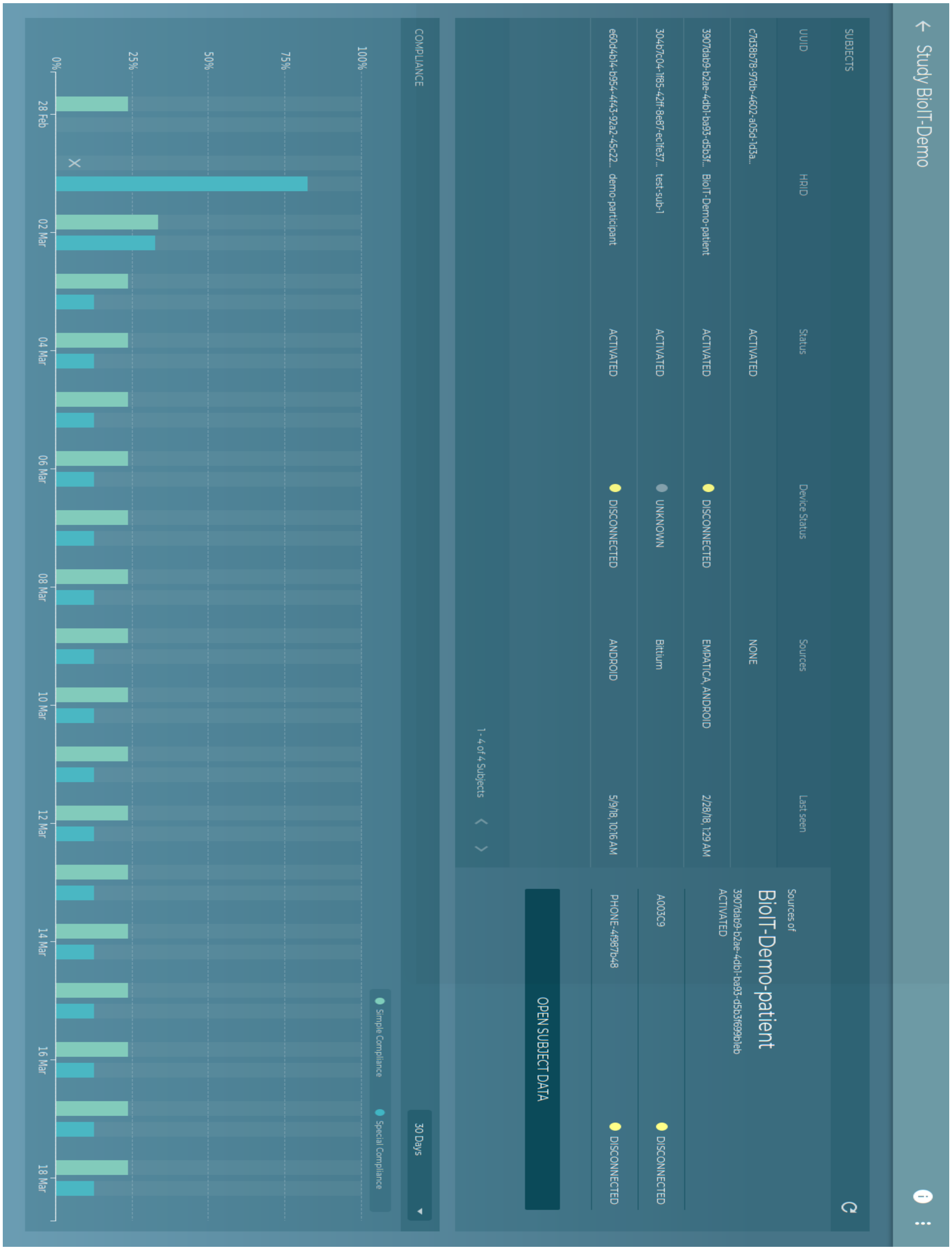

Supplementary Figure 5: Dashboard: Different participants status.

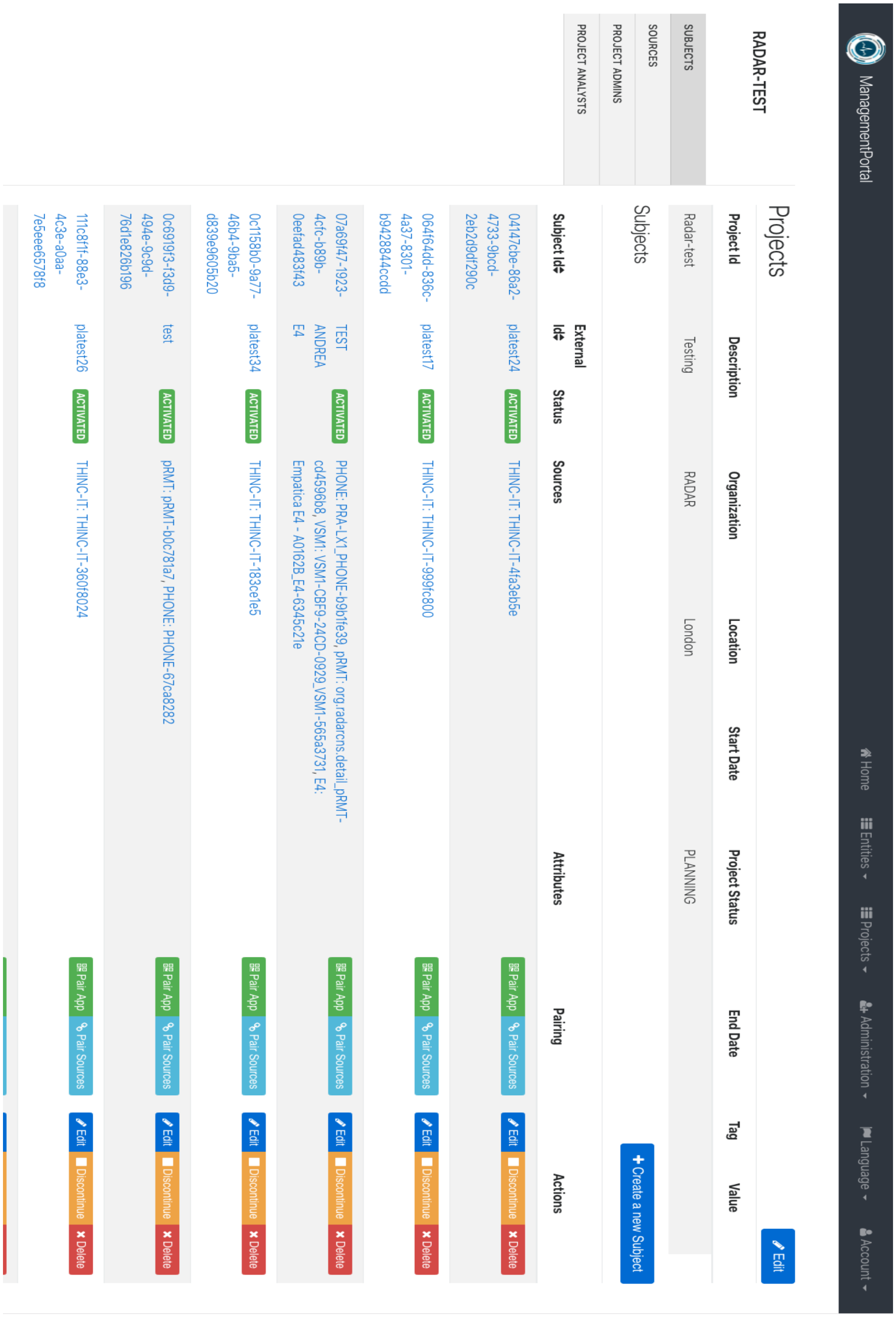

Supplementary Figure 6. RADAR-base Management Portal.
