# Supplementary material for: RADAR-Base: Open Source Mobile Health Platform for Collecting, Monitoring, and Analyzing Data Using Sensors, Wearables, and Mobile Devices
Source: JMIR Mhealth Uhealth. 2019 Aug 1;7(8):e11734. doi: 10.2196/11734 (PMC6694732; doi:10.2196/11734)
Supplement: Multimedia Appendix 2 [file mhealth_v7i8e11734_app2.pdf]

Appendix 1

RADAR-base: An Open Source mHealth Platform for Collecting, Monitoring and Analyzing Data Using Sensors, Wearables, and Mobile Devices

Available sensing platforms:

**Name**	**Creator**	**Software License**	**Open Source**	**Latest Release Date**
Aware	Community Imaging Group, University of Oulu, Tokuda Laboratory, SFC, Keio University	Apache License 2.0	Yes	January 20 2017
Beiwe Research Platform	HSPH Onnela Lab	BSD Licenses	Yes	Dec 21 2017 iOS<br>April 2018 Android
Sage Bridge	Sage Bionetworks	Apache License 2.0	Yes	2018
CenceMe	Smartphone Sensing Group, Dartmouth College		No	July 27 2009 (iOS)
Context Sensing SDK	Intel	Intel EULA	Yes	March 18 2016 (Android)
Emotion Sense	University of Cambridge	BSD Licenses	Yes	April 25 2017
Empath	University of Virginia		No	2011
Expimetrics	Purdue University	Proprietary	No	2018
Funf Open Sensing Framework	MIT Media Lab, maintained by Behavio (Google)	LGPL	Yes	September 4 2015

ResearchKit	Apple	BSD	Yes	June 7 2017
ResearchStack	Cornell Tech's Small Data Lab and Open mHealth	Apache License 2.0	Yes	18 Nov 2016
